# Supplementary material for: Interventions for School-Aged Children with Auditory Processing Disorder: A Scoping Review
Source: Healthcare (Basel). 2024 Jun 7;12(12):1161. doi: 10.3390/healthcare12121161 (PMC11203214; doi:10.3390/healthcare12121161)
Supplement: Supplementary file 1 [file healthcare-12-01161-s001.zip › healthcare-2986605-supplementary.pdf]

Table S1: Characteristics of intervention studies for children who have an APD or AP difficulties

Intervention studies – Auditory training							
			Participant characteristics (including inclusion/exclusion criteria when specified)				
**Authors/ yr/ country**	**Abilities aimed and intervention strategy**	**Study design and follow-up**	**Exp. group**	**Control group**	**Key findings**	**Effect size**	**Generalization and transfer**
Ahmed et al., 2016/ Egypt [1]	Multiple AP abilities: Temporal processing and phonemic awareness	Pre/post Follow-up: N/A.	N = 50 (6 to 12 yrs old; 26 girls) **Gr A and Gr B**: (n=25), subgroups: **1**: n=8, aged 6 to < 8 yrs **2**: n=8, aged 8 to < 10 yrs **3**: n=9, aged 10 to < 12 yrs - NH (threshold below 15 dB HL from 250–8000 Hz); N SRT; excellent speech discrimination; N tymps and ARTs. -AN results on the DDT and PPT - Learning disability, but no neurological disorders and average psycho-intellectual abilities Gr A: formal training; Gr B: informal training	N/A	Interventions were effective at improving dichotic and temporal processing abilities, phonemic awareness and cortical P1 latency. 1) PPT and DDT: Significant improvements for both groups post-training. No statistically significant difference between the 3 age subgroups. 2) Phonemic awareness: Significant improvement in phonemic awareness abilities in both grs. 3) Cortical P1 latency: No statistically significant difference between the three age subgroups, but average latency significantly improved post-training.	Effect size: N/A.	Benefits were extended to other AP abilities (binaural integration).
Oraky et al., 2017/ Egypt [2]	Multiple AP abilities: Temporal processing and phonemic awareness	Pre/post Follow-up: N/A.	N = 50 (6 to 12 yrs old) **Gr A** (n=25, mean age: 8.90 ± 1.73; 14 girls); **Gr B** (n=25, mean age: 8.99 ± 1.67; 12 girls) - APD (test battery included DDT, PPST, AFT); NH (thresholds below 15 dB HL from 250–8000 Hz); N SRT; excellent speech discrimination scores; N tymps and ARTs. - Absence of visual or neurological disorders and average psychointellectual abilities (Arabic Stanford Binnet Test, v.4) Gr A: computerized training Gr B: informal training	N/A	Improvements noted in AP measures, cortical measures and perception following both trainings. Pre- and post-training measures were note statistically different between grs. Statistically significant improvement in: 1) Phonemic awareness 2) APD measures (except AFT at 250Hz, but more improvement for gr A.) 2) P1 latency: Significantly shorter post-training bil. 3) CAPD questionnaire: Results improved significantly.	Effect size: N/A.	Gains were measured on binaural integration and auditory fusion abilities.
Donadon et al., 2019/ Brazil [3]	Multiple AP abilities: Binaural integration, temporal resolution and	RCT Follow-up: N/A.	n=20, 8 to 14 yrs old (10.65 ± 1.56 yrs) - APD (scores below norms for at least 2 tests: DDT, FPT, GIN, SSI-ICM); NH (≤15 dB HL from 250 to 8000Hz); N tymps;	n=14, 8 to 14 yrs (mean 10.85 ± 1.66 yrs) Same characteristics as the exp. gr.	Gr not statistically different before training. 1) DDT, FPT, GIN, SSI-ICM scores: Statistically significant improvements post-auditory training.	Effect size: N/A.	N/A

	ordering, auditory figure-ground		- History of OM, none in the past 12 mos, absence of PE tube;<br>- No history of chronic medical or neurological disease, invasive developmental disorder, learning and/or language difficulties;<br>- Not receiving other intervention.	Completed visual training.			
Filippini et al. (2012)/ Brazil [4]	Multiple AP abilities: SIN, dichotic speech, temporal processing	Non-randomized experimental study Follow-up: N/A.	7 to 13 yrs old (mean = 9.09 ± 1.54 yrs)<br>- NH (thresholds below 20 dB HL from 250 to 8000 Hz); N click-evoked ABR;<br>- Absence of neurological, cognitive, or psychiatric disorders.<br>**Gr APD** (n = 9).<br>- No language or learning disorder<br>- Deficits on 2 AP tests of the APD battery (SIN test, SSW, DDT, PPST)<br>**Gr SLIa** (specific language impairment, n = 6).<br>- Diagnostic of SLI and deficits on at least one of the APD test battery.<br>- Normal overall IQ (PMR).<br>- Had concurrent speech therapy.<br>Both grs: formal auditory training.	Aged 7 to 13 yrs old (mean = 9.09 ± 1.54 yrs)<br>**Children TD** (n = 7) N hearing, language and learning development. Absence of APD<br>**SLIb** (n = 8).<br>- Same characteristics as **SLIa**; underwent speech therapy only.	c-ABR in background noise and AP skills improved following AT.<br>1) SIN test, SSW or DDT, PPST: For APD and SLIa grs, results improved significantly post-training. No change for CGs post-training.<br>2) c-ABR: APD gr had shallower VA slope in silence. No change in silence for any peaks after training. APD and SLIa gr had decreased latencies to c-ABR in background noise after training (SLIa had greater improvement). For peak E, only the SLIa gr had a significantly earlier latency compared to pre-assessment.	Effect size: N/A.	N/A
Schochat et al. 2010/ Brazil [5]	Multiple AP abilities: Frequency, intensity and temporal training, dichotic training, localization and SIN training	Pre/post Follow-up: N/A.	n=30, 8 to 14 yrs old<br>- APD (results below normal in at least one ear for 2 tests: PSI, SIN test, SSW or DDT and NVDT); N ME function; NH (thresholds ≤ 20 dB HL from 250 to 8000 Hz).<br>- absence of neurologic pathology, head trauma, drug use, speech or language problems and normal overall IQ (WISC).<br>- learning difficulties reported by teacher.	n=22, 8 to 14 yrs old Completed informal training at home.<br>- No APD; N ME function; NH; N IQ;<br>- No neurologic pathology, head trauma, drug use, language and learning disabilities, academic difficulties.	Neurophysiologic changes in amplitude and improved AP abilities post-AT.<br>1) PSI, SIN test, SSW, DDT and NVDT: Significant improvement post-training for the exp. gr.<br>2) MLR: No change in the latencies for the treatment gr. following training. Latency measures were not statistically different between both gr. for the 2 testing sessions. Na-Pa amplitude increased significantly post-training for the exp. gr.	Effect size: N/A.	N/A
De Melo et al., 2018/ Brazil [6]	Multiple AP abilities: Auditory figure-ground, dichotic listening, temporal resolution, temporal pattern, localization and auditory discrimination	Comparative, transversal, longitudinal and experimental study Follow-up: N/A.	N=14, between 7 and 8 yrs old<br>**Gr1**: n=7 APD and typical phonological acquisition;<br>**Gr2**: n=7 APD and atypical phonological acquisition<br><u>Inclusion criteria</u>: NH, changes in speech and/or acquired phonological system; APD (failure in at least 1: RGDT, PSI test, NVDT);<br>- Not using regular musical instruments and never completed auditory training.	N/A	Neurophysiological changes were noted following training for both grs. Benefits in functional auditory behaviours were perceived by the parents.<br>1) LLAEP: Post-AT, latency of N2 and P3 in the LE decreased significantly for Gr1 and P2 latency decreased significantly in the RE for Gr2. P3 latency decreased for both grs following training, but only significantly for the LE in Gr1. Some	Effect size: N/A.	Improvement in functional auditory behaviours noted.

			Exclusion criteria: Neurological, emotional and/or cognitive disorder; HL; oral language problems; motor or organic changes.		children had no P3 pre-training, but a measurable one post-training.<br>2) SAB: Statistically significant improvement following AT. Correlations between latencies and SAB scores for both grs.		
Barker and Bellis, 2018/ New Zealand [7]	Dichotic listening (interaural timing difference)	Pre/post, self-control design; assessment twice with 7-14 days interval, pre-training as a within gr control.<br>Follow-up: N/A.	N=15, 8 to 12 yrs old (mean age: 10.3; 7 girls)<br>Inclusion criteria: Struggling academically; NH (screening at 20 dB HL for 500 to 4000 Hz bil.) and N tymps; dichotic deficits (AN result at the DDT in at least one ear); absence of other known diagnosis or disorder.<br>- Not following other therapy.	N/A	No significant difference between the 2 pre-AT scores. Scores for the LE were significantly worse than the RE. Benefits of AT on dichotic listening abilities.<br>1) DDT: Scores significantly improved post-training, especially in the LE. Number of sessions did not correlate with the degree of improvement.	Large to very large effect size (d = 1.94 for the LE and d = 1.15 for the RE).	Children anecdotally reported increased classroom confidence and improved ability to follow multi-step commands at home.
Delphi et al., 2018/ Iran [8]	Dichotic listening (DIID and DOT)	RCT<br>Follow-up: 2 wks after training.	N=12, 8 to 9 yrs old<br>**DIID gr**: n=6 (means age: 8.33 yrs ± 0.51); **DOT gr**.: n=6 (means age 8.50 yrs ± 0.54)<br>- AN result (≥ 2 SDs from established norms) on DDT, PPST, mSAAT and AN REA score; N PTA bil. (thresholds ≤ 20 dB HL from 500 to 4000 Hz) with ≤5 dB HL between 2 ears; N tymps; Wechsler IQ-children score ≥85.<br>- Monolinguals.<br>- No history of ADHD, seizures, behavioural or developmental disorders and not taking CNS medications; poor academic performance.	N/A	DIID and DOT improved dichotic listening and children had N dichotic abilities post-AT.<br>1) DDT: Performance significantly increased LE scores over the course of the trainings. REA WNL post-AT. Dichotic listening improved for both trainings with no significant difference between grs. DOT needed more training sessions than DIID to achieve the same amount of improvement.	Effect size: N/A.	N/A
Mahdavi et al., 2021/ Iran [9]	Dichotic listening (DITD)	Pre/post<br>Follow-up: N/A	N=25, 7 to 12 yrs old (mean 9.3 yrs, SD 1.7 yrs; 7 girls)<br>8 had unilateral DLD and 17 had bilateral DLD.<br>Inclusion criteria: Learning difficulties; DLD (AN results in one of the tests: PRDDT, PCWT, PCST); N tymps, NH (thresholds lower than 20 dB HL from 500 to 4000 Hz); WRS at least 90% bil.<br>- Absence of language problems, medication use, and psychiatric or neurological impairment.	N/A	AT significantly improved dichotic listening of children with DLD.<br>1) PRDDT, PCWT, PCST: Scores post-intervention significantly increased in all dichotic tests for both dominant ear and non-dominant ear.	Hedges's g: large effect size for non-dominant ear and a medium-to-large effect size for dominant ear in dichotic tasks.	N/A
Moncrieff and Wertz, 2008/ USA	Dichotic listening (DIID)	**Pre/post**	**Phase I**: N=8, 7 to 13 yrs old (mean 9.7 yrs, 2 girls) divided in 2 subgroups: **LED and BLD**	N/A	AT improved dichotic listening skills in both ears and performance was more symmetrical post-AT. AT	Effect size: N/A.	**Phase I:** Some children had improved

[10]		**Phase I**: follow-up 1 yr post-training.	7 = speech and language diagnostic; 1 = medicated for ADHD; 7 = no neurological deficit, but 1 = Chiari malformation.<br>**Phase II**: N=13, 6.4 to 11.5 yrs old (5 girls) divided into 3 subgroups: **LED, BLD, WNL**.<br>No diagnosis of ADHD; at risk for language disorder; first-grade level performance on the listening comprehension subtest of the Brigance comprehensive inventory of basic skills-revised.<br>**Phases I and II**: unilateral dichotic deficits; NH (thresholds from 500 to 4000 Hz at 20 dB HL or better; N intelligence.<br>- Significant interaural asymmetry at the DDT (LE 20% poorer than RE for children under 8 yrs old, 15% for 8 to 9 yrs old and 10% for 10+ yrs old).		improved language comprehension and word recognition.<br>**Phase I:**<br>1) LPFS and DDT: BLD gr improved in in LPFS and for RE on DDT. LE DDT was significantly better for both grs post-AT. Pre-training differences between RE and LE scores were no longer significant post-training. Improvements were maintained (n=4) for DDT 1 yr post-training.<br>2) C-TOPP: Phonological awareness increased significantly post-AT for both grs.<br>**Phase II:**<br>1) DDT and CW: Scores improved significantly post-AT. RE and LE scores at DDT no longer significantly different post-AT for subgroups. Post-AT, CW scores were still significantly different between ears, and LE CW improvements were greater overall. **LED** gr and 2 children in the **BLD** gr had performance WNL at the DDT post-training.<br>2) Brigance comprehensive inventory of basic skills-revised: Significant change. **BLD** gr had the largest improvement for listening comprehension and oral reading.		phonological awareness and LPFS.<br>**Phase II:**<br>Improvement in listening and language skills noted for some children.
Nazeri et al., 2020/ Iran<br>[11]	Dichotic listening (DIID)	Pre/post<br>Follow-up: N/A	N=17, 8 to 12 yrs old (mean age: 9.4 ± 1.5 yrs; 11 girls)<br>- Diagnosed with amblyaudia and a learning disability.<br><u>Inclusion criteria</u>: Low scores at the Buffalo Model questionnaire; NH (thresholds from 500 to 4000 Hz at 15 dB or better and asymmetry < 5 dB between ears), N tymps; asymmetry over 25% between the 2 ears in the CWT and RDDT.<br><u>Exclusion criteria</u>: Uncontrolled ADHD and autism.	N/A	DIID training improved dichotic listening.<br>1) P-SSW: Significant reduction in the mean score for DEC, TFM and DEC following training. A reduction in the total mean score of the P-SSW noted post-training.	Effect size: N/A.	N/A
Shoemaker (2010)/ USA<br>[12]	Dichotic listening (interaural time difference)	Pilot study and pre/post<br>Follow-up: N/A	**<u>Pilot study</u>:** n=12, 7 to 12 yrs old (mean age: 9.33)<br>- APD (test battery: SSW, SCAN-C/A, DDT, and SAAT); scores greater than 2 SDs below the mean on SSW and/or	**<u>Pilot study:</u>**<br>n=4, aged 8 to 11 yrs old (mean age: 9.75)<br>**<u>Pre/post:</u>**	DAT with monosyllabic words appears to improve dichotic AP abilities of children with APD.<br>**<u>Pilot study:</u>**	**<u>Pilot study:</u>**<br>Large effect size (partial η2) for 7 of the 19 variables tested.	Recognizing filtered or distorted words improved although it was

			SCAN; NH (thresholds between 0-25 dB HL from 250 Hz-8000 Hz); N ME function; absence of neurological disorders such as autism, mental disabilities or developmental delays; English was primary language.<br>- Children with a diagnosis of ADHD were not excluded.<br>**Pre/post**: n=16, 7 to 12 yrs old (mean age: 9.19; 8 girls). Same characteristics as pilot study.	**CG A with treatment**<br>n=10, 6 to 12 yrs old (mean age: 8.7; 2 girls)<br>**CG B without treatment**<br>n=10, 6 to 14 yrs old (mean age: 9.9; 3 girls)<br>**Both studies:** Same characteristics as exp. gr, but absence of APD.	- No significant differences found between grs on pre-test measures.<br>1) DAT screening tool: Significant improvement for both grs post-intervention.<br>**Pre/post:**<br>1) DAT screening tool: Significant improvement post-AT. Children with APD improved more than CG A. No significant difference noted for CG B.<br>2) CW and FW: Significant improvement for CW LE after DAT. Exp. gr improved significantly on the FW-L compared to CG A. No significant improvement for CG B.<br>3) SSW: Significant improvements in all conditions for exp. gr compared to TD.	**Pre/post**<br>Medium and large effect sizes (partial η2) were noted for some variables of the SCAN-C/A and SSW.	not directly trained by DAT.
Stephenson, 2008/ USA [13]	Dichotic listening (interaural timing difference)	Pre/post<br>Follow-up: N/A	N=8, 7 to 12 yrs old<br>- APD (per test battery including SSW, SCAN-C, and SCANA).<br>- NH (speech and puretone thresholds (0-25 dBHL from 500 to 4000 Hz), N tymps (one child had bil. PE tubes with large ear canal volumes).<br>Exclusion criteria: HL; first language not English; low cognitive function; diagnosed with other disorders (i.e., attention deficit disorder, language impairment, autism).	N/A	Improvement in dichotic listening following DAT.<br>1) DAT screening tool: Some variables improved significantly post-intervention.<br>2) SSW: Number of errors and errors in LC decreased significantly post-intervention.<br>3) SCAN-C/A: No significant differences noted for any of the conditions.	Large effect size for overall score on the DAT (partial η2 = 0.832).<br>Large effect size for number of errors on the SSW and in the LC condition (partial η2 = 0.838 and 0.835)	The skills trained in the DAT did not transfer to other AP abilities (SCAN-C/A).
Lotfi et al., 2016/ Iran [14]	Lateralization	RCT<br>Follow-up: N/A	n=30, 8-12 yrs old (mean age: 9.00 ± 1.28 yrs; 10 girls)<br>Inclusion criteria: Suspected APD (MAPA test battery: results at 2 SDs from the mean in DDT, PPS and mSAAT; N PTA (thresholds < 20 dB HL from 500 to 4000 Hz); PTA difference < 5 dBHL between ears; N tymps; 85 or higher Wechsler IQ score; monolingual (Persian); no history of ADHD, seizures, behavioural or developmental disorders; not been on any CNS medications; poor academic performance.	n=30 (mean age: 9.07 ± 1.25 yrs; 10 girls). Did not follow an intervention. Retested 2 mos after initial assessment. Same inclusion criteria as exp. gr.	Spatial processing and SIN abilities improved post-AT.<br>1) mSAAT: Scores improved significantly bil.<br>2) Spatial WRS in noise at various degrees azimuth improved significantly and the number of lateralization errors decreased significantly. WRS declined significantly for some degrees azimuth after 2 mos for the CG but not for other locations. The number of auditory lateralization errors did not significantly change for the CG.	Effect size: N/A.	N/A
Lotfi et al., 2019/ Iran [15]	Lateralization	RCT<br>Follow-up N/A	See Lotfi et al., 2016	See Lotfi et al., 2016	Binaural processing and SIN perception improved and neurophysiological changes noted post-training.	Effect size: N/A.	N/A

					1) mSAAT: Scores significantly improved bil. for exp. gr, but were stable for the CG.<br>2) BIC: Latency decreased and amplitude increased significantly after AT. No change noted for CG.		
Koravand et al., 2019/ Canada [16]	Singing lessons (targeting pitch and rhythm)	Pre/post<br>Follow-up: N/A	N=11, 7 to 11 yrs old (mean age: 9 yrs, 11 mos; SD: ±1 yr and 3 mos; 3 girls).<br>- APD (2 SDs below the mean on a minimum of 2 tests: SSW, DDT, PPT, RGDT, FWT, BKB SIN/Quick SIN)<br>NH (thresholds ≤ 15 dB HL from 250 to 8000 Hz); N tymps;<br>- Children had concomitant diagnosis/difficulties: learning disability, reading difficulty, apraxia, speech disorder, ocular and motor function difficulty, concerns with attention. One had ASD.<br>-Canadian English-speaking.<br>Inclusion criteria: AN PPT and RGDT.	N/A	Improvement in the magnitude, but not the timing, of several subcortical responses following training.<br>1) click ABR: Post-training, no significant difference for latencies and amplitude.<br>2) cABR: Post-training, no significant difference in neural timing (latencies), but significant difference in neural magnitude (amplitude; VA slopes and peak A significantly bigger). Amplitude of peak O was bigger post-training. 60% of participants had minor amplitude changes for several of the speech-ABR post-training.	Effect size: N/A.	N/A
Tomlin and Vandali, 2019/ Australia [17]	Musical pitch training (pitch discrimination and spectral-timbre variations)	Pre/post: Longitudinal with 2 baseline and 2 post-training assessments.<br>Follow-up: 60 wks post-training (n=5).	N= 10, 7 to 12 yrs old (mean age 10.09 ± 1.49; 2 girls)<br>- Temporal patterning deficit: AN FPT (score below 2 SDs of norms);<br>- Binaural integration deficits (n=2);<br>- NH (15 dB HL or better from 250 Hz–8 kHz range); N tymps; repeatable ABR; non-verbal IQ scores > 85 (TONI-4);<br>- No neurodevelopmental or behavioural conditions.	N/A	The AT could be used to remediate temporal patterning deficits in children. Average training duration: 9.19 (SD 7.04) hrs. Average training period: 110 (SD 70) days. No correlation between degree of change and hrs or duration of training.<br>1) FPT: Baseline measures of FPT significantly correlated. Significant improvement in FPT post-training. No significant difference in late-outcome scores.<br>2) Other: No significant change noted for reading fluency, attention, memory and LIFE-R scores in post-training assessments.	Effect size: N/A.	N/A
Negin et al., 2018/ Iran [18]	Phonemic awareness (phonemic training program)	SSD<br>Baseline: Measures twice a wk for 3 wks.<br>Training: Measures every 2 sessions.<br>Post-Training:	N=1, 9 yr old girl<br>- APD: Scores below the norm for P-PST and P-SSW.<br>Inclusion criteria: NH (thresholds below 15 dB HL from 250-8000 Hz); N ME function; N IQ; N vision; monolingual Persian<br>- Medical treatments and behavioural therapies were continued during the study; history of chronic otitis media and speech development delay.	N/A	Benefits of training for children with binaural integration and phonemic synthesis difficulties.<br>1) P-SSW: Improvements in binaural integration noted (decoding category).<br>2) Phonemic Synthesis Test: No significant change post-training.<br>3) Number of phoneme errors decreased significantly across sessions. Training effects were persistent 2 mos post-therapy.	Effect size: N/A.	Authors report improvement in academic performance and more precisely in spelling skills.

		measures 3 wks after training and every wk for 6 wks.					
Cameron and Dillon (2011)/ Australia [19]	Spatial processing and binaural processing (LiSN & Learn)	Pre/post Follow-up: 3 mos post-training.	N=9, aged 6 yrs, 9 mos to 11 yrs 4 mos (3 girls). - First language is Australian English; - NH (thresholds of 15 dB HL or better from 500 to 4000 Hz and 20 dB HL or better at 250 and 8000 Hz); N tymps; IQ WNL (WISC-IV) (participant 3 scored fourth percentile); no ADHD. Inclusion criteria: Aged 6 to 11 yrs; spatial processing disorder (performance below 2 SDs from the mean on the LiSN-S spatial advantage measure or outside N limits on the LiSN-S pattern measure).	N/A	Average improvement in SRT over the sessions. 1) LiSN-S Test: Significant improvement post-training when speech is spatially separated from distractors. All children scored WNL post-training and scores stable for most at 3 mos post-training. 2) TOVA-A: Only nonsignificant improvement noted for commission errors in the attention test. No change at 3 mos noted. 3) TAPS-3: Significant improvement for memory subtest. No significant difference in performance between both post-training measures. 4) SSQ: Significant improvement post-training in ability to understand speech in quiet and noisy environments.	Large effect size for binaural processing conditions of the LiSN-S when speech and distractor are separated ($\eta 2$ = 0.694, 0.843 and 0.873), memory ($\eta 2$ = 0.571), and SSQ: understanding speech in quiet ($\eta 2$ = 0.708) and understanding SIN ($\eta 2$ = 0.93).	Investigated with attention and memory tests, and the SSQ.
Cameron et al., 2012/ Australia [20]	Spatial processing and binaural processing (LiSN & Learn)	RCT (blinding of participants to their gr assignment) Follow-up: N/A.	n=5, aged 6 to 9; 9 (mean 7; 9; 5 girls) - NH (thresholds 15 dB or better from 500 to 4000 Hz and 20 dB HL or better at 250Hz and 8000Hz); N tymps; SPD (LiSN-S); no reported learning or attention disorder except for one child who had medicated ADHD; absence of non-medicated ADHD; English as first language. -None of the participants used an FM system in the classroom nor undertook other therapy.	n=5 aged between 6;5 and 9;6 (mean 8;4; 5 girls) - Same characteristics as the exp. gr, but one child had mild Asperger's disorder. Completed the Earobics program for 15 min/day for 5 days a wk and for 12 wks.	Average improvement noted in SRT over the course of the training for the exp. gr. 1) LiSN-S: Exp. gr improved significantly on measures when target speech was spatially separated from distractors, but CG did not. No significant improvement for both gr when the target speech was not spatially separated from distractors. 2) LIFE-R: Children and teacher noted improvement in listening skills for both grs, with more improvement the exp. gr. 3) FAPC: Parents noted significant improvement in AP behaviours post-training for the exp. gr only.	Very large effect size in the high cue SRT ($\eta 2$ = 0.855), in the spatial advantage measure ($\eta 2$ = 0.954), and in the total advantage measure ($\eta 2$ = 0.752) Very large effect size ($\eta 2$ = 0.803) for Fisher's (listening skills perceived by parents).	N/A
Graydon et al., 2018/ Australia [21]	Spatial processing and binaural processing (LiSN & Learn)	Pre/post Follow-up: mean 10 ± 5 mos post-AT (n=13).	N=16, aged 6; 3 to 10; 0 (mean 7; 8 ± 1; 2; 7 girls) - NH (Thresholds at 20 dB HL or better from 500 Hz to 8000 Hz); N tymps; No speech/language delay and no	7 participants re-tested to confirm the SPD diagnosis, which provided within subject control.	Average number of completed games 85 ± 31 (range 20 to 146). 9 participants did not complete 100 games due to lack of motivation. The mean pattern z-score pre-training was - 2.7 ± 0.5 for the gr.	Effect size: N/A.	Significant improvements noticed in real life situations per parents.

			cognitive impairment; SPD (LiSN-S pattern z-score ≥ 2 SDs from mean).		1) LiSN-S: No significant difference between pre-testing measures. Significant improvement noted following intervention for measures with spatial cues and DV0 condition. No difference noted for talker advantage and SV0 condition. Improvements 10 mos post-training. 2) LIFE, FAPC and TEAP: All had listening difficulties pre-training. Improvement in all 3 questionnaires noted post-training, but significant per the parent only.		Improvements noted at school per children and teachers.
Brasil and Schochat, 2018/ Brazil [22]	Speech-in-noise (*Programa de Escuta no Ruído* [PER])	Pre/post, self-control design; 1) Pre-training evaluation 2) 12 wks: post-placebo intervention assessment of AP skills 3) Post-PER assessment of AP skills Follow-up: N/A.	N=18, 8 to 10 yrs, 13 boys (mean 9; 7) and 5 girls (m 9;0). <u>Inclusion criteria</u>: APD; absence of peripheral auditory disorder; NH; N ME function; absence of motor impairments, cognitive and speech motor development impairments, neurological damage, restricted social interaction, and significant emotional disturbances; poor school performance due to learning difficulties.	N/A	No significant difference between the 2 pre-intervention measures of AP. AP skills improved following PER training. Benefits of PER on reading, writing and arithmetic abilities. 1) SIN, PSI, SSW, FPT: Results indicate significant differences between the pre- and post-auditory training conditions for AP measures. 2) SAT: Slight, but not significant, difference between the pre- and post-AT conditions in the SAT.	Large effect size (η2partial = 0.905) in behavioural measures of AP. Small effect size(η2partial = 0.372) in SAT.	Some improvement noted for academic skills, such as writing, reading and arithmetic (SAT).
Jutras et al., 2015/ Canada [23]	Speech-in-noise (*Logiciel d'écoute dans le bruit* [LEB])	Pilot study Follow-up: 6 mos post-training.	n=5, 8 to 12 yrs old (mean 10) with APD. <u>Inclusion criteria</u>: NH (threshold ≤ 15 dB HL from 250–8000 Hz); APD (failure at 2 SDs from the mean in at least one ear, on 2 of the AP tests: dichotic listening, SIN, and temporal processing); failure in at least one SIN test or dichotic test; French main language used at home and school; absence of attentional or neurological problems reported by parents.	n=5, aged 8 to 12 yrs old (mean 10; 6) with APD. Did not receive therapy.	Trends were discussed. 1) Children were more tolerant to noise level as therapy progressed. The percentage of correct responses improved while noise levels increased. 2) HINT: No change noted compared to CG following training. 3) P1-N1-P2-N2: Post-training, P1 latency was for the exp. gr, but not the CG. N2 amplitude increased more for the exp. gr than for the CG. P1 latency did not change 6 mos post-training. N2 amplitude increased 90% for the exp. gr 6 mos post-training, but increased 10% for the CG. 4) ECA, MHAVIE, Sifter (teacher only): No changes noted by teachers and parents post-training.	Effect size: N/A.	Social impact investigated with the MHAVIE questionnaire. No change noted immediately after and 6 mos post- training.
Jutras et al., 2019/ Canada	Speech-in-noise	Exploratory study, Pre/post	n=10, 8 to 12 yrs old (mean: 10 yrs, 6 mos; SD: 6 mos)	n=6 (mean age 9 yrs, 10 mos, SD: 13 mos)	1) Children were more tolerant to noise level as the therapy progressed (noise levels increased significantly	Large effect size for performance across therapy as	No change was noted immediately

[24]	(*Logiciel d'écoute dans le bruit* (LEB))	Follow-up: 3 mos post AT.	Inclusion criteria: NH (threshold ≤ 15 dB HL from 250–8000 Hz); APD (failure at 2 SDs from the mean in at least one ear, on 2 AP tests); failure in at least one SIN test or dichotic test; French is the main language spoken at home and school; absence of attentional or neurological problems reported by parents.	Same characteristics as exp. gr.<br><br>2 children followed the therapy after control measures.	from session 6-24 with no change in percentage of correct responses).<br>2) HINT: No significant improvement noted in the training gr compared to the CG. More children in the training gr improved at the HINT than the CG.<br>3) P1-N1-P2-N2: No significant between-gr differences, but P1 and N2 latencies and amplitudes indicated trend for positive neurological changes in the exp. gr.<br>4) SAB, SIFTER: No significant changes following training per teachers, but individual data shows the exp. gr improved in its ability to discriminate and identify speech and to understand rapid or muffled speech.	noise levels increased (η2 = 0.453 for percentage of correct response and η2 = 0.868 for SNR).	after and 6 mos after the training in auditory behaviours.
Loo et al., 2012/ Singapore [25], [26]	Speech-in-noise	Prospective RCT (teachers blinded to grs).<br>Follow-up: 3 mos post AT.	n=20, 7 to 11 yrs old (average 9,1 yrs, SD 1.33)<br>-2 children had medicated ADHD<br>Inclusion criteria: Being in mainstream school; listening difficulties, NH (thresholds of 20 dB HL or better from 250 Hz to 8 kHz), N tymps, ipsi ART at 1 kHz < 100 dB HL; speech discrimination scores in quiet ≥ 80%; AN scores (2 SDs criteria) in ≥ 2 (but not all) APD tests: FPT, DPT, RGDT, RGDT, MLD, DDT); N nonverbal IQ (> 85 TONI-3); absence of autism, and neurological conditions.<br>Some children had auditory memory, phonological or language difficulties.	n=19, 7 to 11 yrs old (average 9 yrs, SD 1.32)<br>-2 children had medicated ADHD and some children had auditory memory, phonological or language difficulties.<br>Same characteristics as exp. gr.<br>Received standard treatment for APD: Listening and educational strategies at school or at home.	Training improved SIN abilities and parent and teacher's perceptions of listening and communication skills. Median of 27 hrs of training completed (range 9 to 30 hrs).<br>1) LiSN-S: Significant improvement in SRTs for the exp. gr post-training. Mean SRT was slightly better at 3 mos post-intervention compared to immediately after. Only total advantage improved significantly, for the intervention gr. No correlation between language and phonological skills, nonverbal IQ and auditory memory and the changes in the overall LiSN-S performance.<br>2) Pragmatic profile and CHAPS: Improvements were greater (and significant) in the exp. gr than the CG. Changes in the LiSN-S were predictors of change in the CHAPS.	Large effect size for LiSN-S scores (Cohen's d = 1.7) and for pragmatic skills (Cohen's d = 1.0). Medium effect size for CHAPS (Cohen's d = 0.76).	AT benefits appeared to generalize to better listening in the classroom environment.
Hassaan and Ibraheem, 2016/ Egypt [27]	Speech-in-noise (auditory figure-ground (AFG)/ noise-desensitization)	Pre/post<br>Follow-up: N/A.	N=17, 7 to 14 yrs old, (mean: 11.9, SD 0.7; 7 girls)<br>- AFG deficits only (n=10) or with other AP deficits; NH; N tymps.<br>Exclusion criteria: Chronic medical or neurological illness, pervasive developmental disorder; not attending school.	N/A	AFG ability, other AP abilities and auditory cortical processing improved following training.<br>1) Arabic SPIN, CST, DPT: Statistically significant improvement on all measures post-intervention.<br>2) Speech-evoked P1-N1: Statistically significant improvement noted for the thresholds in noise measurement of	Effect size N/A.	N/A

					the P1-N1 complex. Amplitude significantly improved post-training. No significant differences for P1 and N1 latencies.		
Jalilzadeh Afshari et al., 2022/ Iran [28]	Speech-in-noise (auditory spectro-temporal modulation [STM])	RCT Follow-up: 1 mo post-training.	n=17, 8 to 12 yrs old (mean: 10.11 ± 1.45; 10 girls) <u>Inclusion criteria</u>: NH (thresholds < 20 dB HL from 500 to 4000 Hz, thresholds difference < 10 dB HL between ears); N typms; ≥ IQ of 85 (Wechsler); monolingual Persian; no history of ADHD, seizures, behavioural or developmental disorders; no medications affecting CNS; poor educational performance; AN result in all 3 subtests of MAPA: DDT, PPS and mSAAT; AN results in STMs detection tasks and SIN tests (CVN and WIN).	n=18, 8 to 12 yrs old (mean: 9.88 ± 1.36 yrs; 11 girls) Same characteristics as exp. gr.	The AT was effective in improving the detection of STM and in improving SIN. Improvement remained 1 mo following training for STMs and CVN, but not WIN test. 1) STM thresholds: Significant differences between training and CG post-training. No change one mo post-intervention. 2) CVN and WIN (Persian): SIN results show significant differences post-training for exp. gr compared to CG. CVN results remained stable one mo post-AT. SNR in the WIN test did not improve or remain stable one mo post-training.	Effect size, N/A	N/A
Kumar et al., 2021/ India [29]	Speech-in-noise (noise desensitization)	Pre/post Follow-up: N/A.	n=10, 9 to 10 yrs old (mean: 10.1 yrs; 4 girls) - NH (thresholds < 15 dB HL from 250 to 8000 Hz); speech identification scores > 80%; N tymps; ispi ART at 1kHz and 2kHz < 100 dB HL; APD (score 3 SDs from the mean at one test or scores at 2 SDs from the mean on 2 tests: GDT, DCV, DPT, SPIN-IE, RAMST); score 2 SDs below mean at SPIN-IE; no language deficits.	n=10, 9 to 10 yrs old (mean: 10.2 yrs; 4 girls;) Same characteristics as exp. gr. - Did not receive intervention, but were invited to SIN training following control measures.	Speech-in-noise training has improved temporal auditory skills, SIN skills and working memory skills. No difference in scores between initial and follow-up auditory processing assessment and cognitive skills for control gr. 1) GDT, DCV, DPT, SPIN-IE, RAMST: Significant improvement for exp. gr post-training, except RAMST and DCV. 2) Digit span test (auditory memory): Exp. gr had significant improvement in all cognitive skills, except forward digit span following intervention. No correlation found between AP skills and memory skills. No correlation between SPIN-IE scores and the other auditory processing measures or cognitive scores.	<u>(r) values</u> Large effect size for SPIN RE (r = 0.63) and LE (r = 0.62), GDT RE (r = 0.62) and LE (r = 0.62), DPT RE (r = 0.59) and LE (r = 0.54). Medium to large effect size for backward digit span (r = 0.46), ascending digit span (r = 0.56) and descending digit span (r = 0.48).	AT had impacts on working memory skills.
Kumar et al., 2021/ India [30]	Speech-in-noise (noise desensitization)	RCT Follow-up: N/A.	n=10, 9 to 13 yrs, M = 11.1 yrs) - NH (thresholds < 15 dB HL from 250 to 8000 Hz); speech identification scores > 80%; N tymps; ispi ART at 1 kHz and 2 kHz < 100 dB HL; bil. N morphology and absolute latencies on click ABR; absence of language, reading, and cognition deficits; APD	n=10, 9 to 13 yrs, M = 11.4 yrs) Characteristics are the same as exp. gr. - Did not receive intervention, but were invited to SIN	AT improved auditory skills, such as SIN and temporal processing, and cortical evoked potential. CG had no significant difference between the 2 evaluations. 1) GDT, DCV, DPT: Significant improvement for the SIN test bil., GDT	<u>(r) values</u>: Moderate effect size for measures of APD and ALLRs that were significantly better following training.	Reduced stress at school after the training was reported. Teachers reported that the children followed

			(score 3 SDs from the mean at one test or scores at 2 SDs from the mean on 2 tests: GDT, DCV, DPT, SPIN-IE, RAMST); score 2 SDs below mean at SPIN-IE.	training following control measures.<br><br>n=7 TD completed electrophysiological measures once.	bil., DPT bil. post-training. No difference noted for DCV.<br>2) RAMST: No difference noted for auditory memory and sequencing.<br>3) SPIN-IE: All participants improved post-training. For the RE, 7 participants had scores within 1 SD of the mean following training and 9 for the LE.<br>4) P1-N1-P2-N2: Following training, P1 N2 amplitudes significantly decreased in quiet and all four peak amplitudes decreased significantly in noise. No change in amplitude and latencies were noted for the CG for both quiet and in noise measures.<br>5) Subjective feedback: Parents did not report improvement in listening skills following training, but participants and teachers did. Reduced stress reported by participants, more responsiveness to instructions reported by teachers.		directions more easily following the training.
Maggu and Yathiraj, 2011/ India [31]	Speech-in-noise (noise desensitization)	RCT (preliminary study)<br>Follow-up: N/A.	n=5, 8 to 11 yrs<br>- Enrolled for at least 5 yrs in schools where the instructions are English and spoke English fluently; NH (thresholds at 15 dB HL or better from 250 Hz to 8000 Hz); N tymps; ART present at 90-100 dB HL; speech identification scores > 90%; absence of speech and language problems; IQ between 90 and 110 (RCPM)<br><u>Inclusion criteria:</u><br>- Failed SCAP; scored lower than 50% on the Monosyllable Speech Identification Test in English for Indian children (SIN test).	n=5, 8 to 11 yrs<br>Same characteristics as exp. gr.<br>Did not receive the intervention, but were offered to receive it after the end of the study.	Noise desensitization training can improve SIN skills.<br>1) SIN: Exp. gr had significantly better scores on all 3 the SIN tests (monaural monosyllables, sound field speech discrimination and sentence identification) compared to the CG following AT. There was no significant difference in performance for the 2 grs at baseline and the CG had no significant difference between the 2 assessments.	Effect size, N/A.	N/A
Maggu and Yathiraj, 2010/ India [32]	Temporal patterning	RCT (CG retested 3 to 4 wks after baseline.)<br>Follow-up: 1 mo post-testing (n=2).	n=5, 8 to 13 yrs<br>- Enrolled for at least 5 yrs in schools where the instructions are in English and spoke English fluently; NH (thresholds at 15 dB HL or better from 250 Hz to 8000 Hz); N tymps; ART present at 90-100 dB HL; speech identification scores > 90%; absence of speech and language problems; IQ between 90 and 110 (RCPM).	n=5, 8 to 13 yrs<br>Same characteristics as exp. gr.<br>Did not receive the intervention.	Temporal pattern training improved temporal patterning and auditory memory and sequencing.<br>Scores were stable across the 2 first evaluations for the CG. Scores for the exp. gr and CG were not statistically different at baseline.<br>1) SPIN, DCV, DPT, GDT:	Effect size, N/A.	Benefits from the temporal pattern training generalized to other skills (auditory memory and sequencing).

			<u>Inclusion criteria:</u><br>- Failed SCAP; poor score on the DPT.		Significant improvement post-training for the exp. gr on the DPT, but not on the other measures.<br>2) RAMST: Significant improvement noted for auditory memory skills.<br>Scores remained constant for both grs when retested one mo post-evaluation.		

INTERVENTION STUDIES – ASSISTIVE DEVICES							
			**Participant characteristics (including inclusion/exclusion criteria when specified)**				
**Authors/ yr/ country**	**Intervention strategy**	**Study design and follow-up**	**Exp. group**	**Control group**	**Key findings**	**Efficacy**	**Generalization and transfer**
Stavrinos et al. (2020), Stavrinos (2019)/ UK [33], [34]	Remote microphone hearing aids (RMHAs), binaural fitting	**<u>Studies 1 and 2</u>**: RCT<br>**<u>Study 3</u>**: Meta-analysis of studies 1 and 2<br>(**Gr A**: APD and no RMHA<br>**Gr B:** APD and RMHAs)<br>Follow-up: N/A.	**Gr B**<br>**<u>Study 1:</u>**<br>n=8, aged 7 yrs, 9 mos to 11 yrs, 1 mo (mean 9 yrs and 6 mos, SD 15 mos; 4 girls).<br>**<u>Study 2:</u>**<br>n=13 aged 7 yrs, 3 mos to 11 yrs, 5 mos (mean 9 yrs and 7 mos, SD, 16,1 mos; 4 girls).<br>**<u>Study 3:</u>**<br>n= 21, mean age: 9 yrs and 7 mos, SD 1 yr and 3 mos; 8 girls).<br><u>Inclusion criteria</u>: Parents reported SIN and listening difficulties, NH (PTA below 20 dB from 250 Hz to 8 KHz); N ME functions; AN results on AFG subtest of SCAN-3 C; 2 SDs from the mean on at least one AP test (DDT, DPT or FPT, GIN or RGDT), or a score of 3 SDs from the mean on one AP test or AFG and/or AN performance (2 SDs below the mean) on the spatial advantage and total advantage/ high-cue SRT conditions of the LiSN-S; absence of neurological or pervasive disorder, or developmental delay; nonverbal IQ > 85 (WNV scale of ability);	**Gr A**<br>Same inclusion criteria as Gr B<br>**<u>Study 1:</u>**<br>n=9, aged 7 yrs, 6 mos to 11 yrs, 8 mos (mean 9 yrs and 7 mos, SD 18 mos; 6 girls).<br>**<u>Study 2:</u>**<br>n=13, aged 7 yrs, 5 mos to 11 yrs, 7 mos (mean 9 yrs and 8 mos, SD 16,3 mos; 4 girls).<br>**<u>Study 3:</u>**<br>n= 22, mean age of 9 yrs and 9 mos, SD 1yr and 5 mos; 10 girls.	**<u>Study 1: Measures: Pre-fitting, 3 mos post-fitting</u>**<br>1) No significant change in intervention gr for SIN (AFG), attention abilities (TEACh) and memory (AWMA) post-intervention.<br>2) CCC-2, CHAPPS:<br>Parents noted improvement in attention at home, but no significant changes in language abilities post-intervention.<br>3) SIFTER: excluded from analysis (low return rate from teachers).<br><br>**<u>Study 2</u>: <u>Measures: pre-fitting, 3 mos post-fitting and 6 mos post-fitting</u>**<br>Usage time was between 60 and 580 hrs and 0,5 to 6.2 hrs/day.<br>1) LiSN-S: No improvement noted for SIN conditions in LiSN-S and spatial listening conditions of the LiSN-S.<br>2) TEACh: The difference between the 2 grs was not statistically significant at 6 mos.<br>3) CHAPPS and CCC-2: no change in scores at the CHAPPS (noise, multiple inputs and auditory attention span) and the CCC-2 (composite score for language).<br>3) LIFE-R: Some questions at the LIFE-R and the total score showed significant improvement in classroom listening situations at 3 and 6 mos of fitting.<br><br>**<u>Study 3</u>: Pre-fitting, 3 mos post-fitting**	**<u>Study 1 and 3</u>**: effect size, N/A.<br><br>**<u>Study 2:</u>** For the total score of LIFE-R, a large effect size was noted (partial $\eta2 = 0.408$) and large effect size noted for Competing speech and traffic noise (partial $\eta2 = 0.306$ and 0.481, respectively).	Some improvement noted by the children in their daily listening situations a school.

			aged between 7 to 12 yrs old; native English speakers. <u>Exclusion criteria</u>: Have used RMHAs in the past.		1) TEACh: Difference between grs was not statistically significant at 3 mos. 2) CHAPS (noise, multiple inputs, and auditory attention span): No change over the 3-mo intervention period. 3) CCC-2 (i.e., standard language composite score): No change over the intervention period of 3 mos. No improvement in attention abilities after 3 mos of wearing the RMHA. Parents did not perceive improvement in language, listening in noise, listening with multiple inputs and auditory attention span after 3 mos.		
Umat et al., 2011/ Malaysia [35]	FM system (personal, monaural or binaural fitting)	Pre/post, longitudinal Follow-up: 1 yr without the FM (n=10).	**Gr 1**: Unilateral-fitting FM (RE) n=19, aged 7 to 9 yrs old (mean 7.95, SD 0.97) **Gr 2**: Bilateral-fitting FM n=19, aged 7 to 9 yrs old (mean 7.89, SD 0.94) <u>Inclusion criteria</u>: NH (screening at 20 dBHL from 500-4000 Hz); N tymps; N IQ; failure (2 SDs below N) in one of the APD screening tests: Malaysian DDDT or PPST. <u>Exclusion criteria</u>: Poor record on school attendance; symptoms of ADHD.	n=15, aged 7 to 9 yrs old (mean 7.93, SD 0.88). Same inclusion criteria as exp. gr.	<u>**Measures: Pre-fitting, 12 wks and 1 yr after not wearing the FM system**</u> 1) RAVLT: At pre-fitting, scores at the RAVLT were not significantly different between the 3 grs. For WM, scores were significantly higher at post-fitting and long-term compared to pre-fitting. No significant difference noted between the 2 exp. grs at post-fitting, but improvements increased for unilateral gr in the long-term. For best learning, long-term mean scores were significantly higher from the mean score obtained at pre-FM system and the difference in performance in Grs 1 and 2 was not significant. For the retention of information, long-term mean scores were significantly higher than at pre-FM, but between-gr mean scores were not significant. Improvements noted in WM (post-fitting and long-term), best learning and retention of information (long-term).	Effect size, N/A.	The use of the FM system can improve short-term auditory memory abilities.
Smart et al., 2018/ New Zealand [36]	FM System (personal, binaural fitting)	Baseline-control design; Visit 2: 8 wks after visit 1. Fitting at visit 2. Visit 3: 5 mos (20 wks) after fitting of FM. Follow-up: N/A.	N=28, aged 7 yrs 3 mos to 12 yrs 9 mos (mean 9 yrs 6 mos, SD 1 yr 7 mos; 6 girls) - APD (scores 2 SDs below the mean on 2 tests or 2 ears of a single test, or 3 SDs below the mean on one test or one ear: FPT, DDT, GIN, CRW (monaural low redundancy); -CELF-4 (forward digit span): standard scores ranged from 3 to 14 (mean = 7.96, SD = 2.85). 9 in APD gr had digit span scores > 1	N/A	Participants assessed with and without FM system. No changes for CRW, MLD, sustained attention scores (IVA-CPT). No significant change for GIN, DDT and SSN hard words. 1) SSN, FPT: For each visit, improvement in SSN scores noted during testing when participants wore the FM system, suggesting immediate benefits of the FM systems on SIN perception. SSN easy words scores improved significantly between visits 2 and 3. FPT and SSN easy words showed stable	Effect size N/A.	Improvement noted by the children and their teacher in their daily listening situations a school.

			SD below the mean; average 7 to 12 yrs old readers per Wheldall Assessment of Reading Passages;<br>APD only (n=7), Asperger syndrome (n=4), learning or language disability (n = 16), ADHD (n=8).<br><u>Inclusion criteria:</u><br>- NH (thresholds 250 to 8000 Hz at 15 dB HL); N tymps and present ART; suspected APD.<br><u>Exclusion criteria:</u><br>- Scores < 80 at TONI.		baseline scores and statistically significant improvements after the FM intervention.<br>2) P1-N1-P2-N2: CAEP analysis did not show changes in amplitudes or latencies that could be attributed to the use of the FM system. P1 and N2 were longer and N2 amplitude were smaller when measured in noise compared to quiet without the use of the FM, but this difference was reduced when wearing the FM. P1 and N2 latencies and N2 amplitude were less affected by noise when the FM system was used.<br>3) LIFE-UK: Significant improvements in children's and teachers' ratings of classroom listening abilities after the FM trial.<br>4) Subjective opinion: The majority of teachers rated the use of the FM as successful and highly successful. Parents noted an overall improvement.		

INTERVENTION STUDIES – HEARING AIDS

Authors/ yr/ country	Intervention strategy	Study design and follow-up	Participant characteristics (including inclusion/exclusion criteria when specified)<br>Exp. group	Participant characteristics (including inclusion/exclusion criteria when specified)<br>Control group	Key findings	Efficacy	Generalization and transfer
Kuk et al., 2008/ USA [37]	Mild gain binaural hearing aids	Pre/post; single-blind, longitudinal descriptive study.<br>Follow-up: N/A.	n=14, 7 to 11 years old (mean 9 yrs, SD 1.4 yrs; 6 girls)<br>- NH (thresholds < 10 dBHL from 500 Hz to 4000 Hz); N tymps and ART pattern; APD (performance below 2 SD of the mean on 2 or more tests: SSW, PS, FW, DDT, CS, PPS, SIN test); N intelligence.<br>- 5 participants had ADHD diagnosis (4 medicated).<br>- All were receiving speech and language therapy at the time of the study and one was also using an FM system in the classroom.	N/A	Almost all children wore their HA in at least some of their daily environments. Half of the children wore the HA consistently at home. Almost all of them wore the HA consistently at school. The majority did not change the programs in their HA, leaving it on their preferred setting.<br>1)SIN: Amplification alone (microphones in omni) did not improve SIN compared to unaided condition. When NR was activated with omni or directional microphones, performance in noise was significantly better, especially for the directional mic and NR. Performance did not improve over the months.<br>2)ACPT: Performance for attention in noise improved slightly (not significantly) over time. Variability in performance was greater in unaided noise condition than in aided noise conditions.<br>3) CHAPPS: Teacher noted improvement in noise and quiet listening situations. Parents	Effect size N/A.	Personal amplification might improve everyday behaviour of children with APD.<br>4 children reported better grades.

					noted improvement in memory and attention tasks.<br>4) Subjective feedback: All parents gave positive feedback following trial.		

INTERVENTION STUDIES – TOP-DOWN TRAINING

Authors/ yr/ country	Intervention strategy	Study design and follow-up	Participant characteristics (including inclusion/exclusion criteria when specified) Exp. group	Control group	Key findings	Efficacy	Generalization and transfer
Moossavi et al., 2015/ Iran [38]	Working memory	Non-randomized case-controlled trial<br>Follow-up: N/A.	n=15, 9 to 10 yrs old (mean age: 9.1 yrs, SD = 0.35); 3 girls)<br>- NH; N tymps; N speech discrimination scores; N IQs (≥85 on Wechsler's scale); no history of neurological disease or injury; APD: MAPA (DDT, PPST, mSAAT); Failure to DDT, PPST and mSAAT<br><u>Exclusion criteria</u>: History of hearing impairment, ear diseases and neurological difficulties.	n=10 (mean age: 9.45 yrs); 3 girls<br>Did not follow training, but were offered to complete it after the study.	Improvement in WM and auditory stream segregation noted post-training.<br>1) CMAA: Significant improvement in the auditory stream segregation tasks after training.<br>2) Persian non-word repetition test and digit span subtests of the Wechsler: Statistically significant improvement for the 3 measures of WM for the exp. gr, but not for the CG.	Large effect size for all 3 reference locations on CMAA: $\eta 2 = 0.45$ for CMAA0°, $\eta 2 =$ 0.71 CMAA30°, and $\eta 2 = 0.58$ for CMAA60°).	N/A

INTERVENTION STUDIES - MULTIMODALITY

Authors/ yr/ country	Intervention strategy	Study design and follow-up	Participant characteristics (including inclusion/exclusion criteria when specified) Exp. group	Control group	Key findings	Efficacy	Generalization and transfer
Bellis & Anzalone, 2008/ USA [39]	FM system; classroom modifications; accommodation strategies; use of clear speech; central resources, attribution, and auditory (discrimination and phonological	Case report<br>Follow-up: N/A.	n=1, 8 yr old boy with reading and spelling difficulties, difficulty hearing in noise, often mishears what others say. Language and speech WNL, but some weaknesses in vocabulary, past history of phonological disorder. Medical history unremarkable, except for a history of ME infections.<br>-NH; N tymps and DPOAEs; excellent word discrimination scores; N ART; APD: deficits	N/A	Restest conducted 3 mos following first evaluation.<br>1) LPFS: Improvements were noted, suggesting that the child's auditory closure abilities improved.<br>2) CST and DDT: Improvements were noted, suggesting that auditory separation and integration abilities improved.	Effect size: N/A	The child's parents and teachers reported a significant improvement in the child's ability to understand SIN in the classroom.

	awareness) trainings.		noted at the DDT (RE), CS (RE), LPFS (bil.). Results on the FPT and DPT WNL.				
Sharma et al., 2012/ Australia [40]	Assistive device (personal FM system, binaural fitting), discrimination and language interventions.	RCT CG: tested twice with 6-7 wks period in between. Follow-up: N/A.	4 exp. grs:<br>1) Discrimination training: n=12 (mean age: 9.8 yrs, SD: 1.6 yrs; 7 girls)<br>2) Discrimination training + FM system in classroom: n=10 (mean age: 10.5 yrs, SD: 1.5 yrs; 3 girls)<br>3) Language training: n=12 (mean age: 9.6 yrs, SD: 1.6yr; 2 girls)<br>4) Language training + FM system in classroom: n=12 (mean age: 10.1 yrs, SD: 1.1 yrs; 6 girls)<br>- APD (performance on any task 2 SDs below the mean on any tests: DDT, FPT, RGDT, compressed and reverberant HINT, MLD). Majority of participants had comorbidities (reading or language disorder) (Sharma et al. (2009).<br>Inclusion criteria (Sharma et al., 2009): N tymps; Score ≥ 80 TONI; NH (thresholds ≤ 15 dB HL); suspected APD or APD.	n=12 (mean age: 8.8 yrs, SD: 1.3 yrs; 4 girls) with APD (same test battery as exp. grs.) Did not receive any intervention.	No differences were noted for pre and post measures of the HINT and CASL for any gr. Performance of CG did not change when retested.<br>1) Discrimination training grs: In discrimination only, significant improvement noted for FPT, concepts and following directions, sentence recall, receptive language and core language (CELF-4). Significant improvement for core language (CELF-4) and syllable segmentation (QUIL) for discrimination + FM gr.<br>2) Language training grs: Improvement for FPT, language (CELF-4 for formulating sentences and core language), phonemic skills (QUIL spoonerism) and reading (WARP) for language only gr. Significant improvement for language (CELF-4 for sentence recall) and phonemic skills (QUIL for nonword spelling) for FM gr.<br>3) Overall, improvement noted for both types of training (discrimination and language) and FM provided an additional benefit over training alone. Benefits found for language, AP and phonological awareness.	For both discrimination grs.: large effect size for core language in the CELF-4.<br>For both language grs.: Large effect size for sentence recall (CELF-4) and nonword spelling (QUIL)	According to anecdotal remarks from parents, and participants real life benefits were observed, such as the children being better able to follow instructions, increased interest in reading, and greater responsiveness and success in class.
Sharma et al., 2014/ Australia [41]	Assistive device (personal FM system, binaural fitting), discrimination and language interventions.	RCT (2 baseline measures and one post-training for APD grs) Follow-up: N/A.	Same exp. grs as Sharma et al. (2012).	1) Same CG as Sharma et al. (2012)<br>2) TD gr (completed CAEP once): n=22, aged 7 to 12 yrs old (mean 10.7 yrs, SD 1.7; 12 girls); no listening or learning difficulties; NH (thresholds ≤ 15 dB HL); N tymps; present ART; N scores for DDT and FPT.	-Children who received interventions had significantly different results in quiet, but not in noise, post-intervention. APD CG did not show significant difference between the measures of visits 2 and 3. N250 amplitude decreased during baseline measures (visits 1 and 2) for both quiet and noise conditions, but not P1 and N250 latencies. Variability was greatest in quiet condition.<br>-Discrimination treatment gr showed a significant change in N250 amplitudes in quiet and noise. Trends were noted for discrimination + FM gr and language gr.<br>- Discrimination training might have a greater impact on CAEP amplitudes than top-down language training.	Effect size, N/A.	N/A
Putter-Katz et al.,	Bottom-up: Auditory training for SIN	Pre/Post with CG	n=20, aged 7 yrs and 11 mos to 14 yrs and 4 mos (mean 9 yrs and 4 mos).	n=10, aged 6 yrs and 2 mos to 11 yrs and 11 mos	Improvements in auditory function (SIN and dichotic abilities) were noted following the bottom-up and top-down intervention.	Effect size, N/A.	N/A

2008/ Israel [42]	and attention, and FM system; Top-down: Coping strategies, accommodation at home, speech reading.	Follow-up: N/A.	2 subgroups:<br>**Noise gr**: n=11 difficulty in SIN task, but N results in competing tasks.<br>**Noise + dichotic gr**: n=9 difficulties in both SIN and competing tasks.<br>Inclusion criteria: Native Hebrew speakers; listening, language, attention, or learning difficulties; NH, excellent word recognition; N ME function; N ABR; N ART; N IQ; absence of psychological or emotional difficulties and neurological deficits; APD (performance at least 1 SD below the mean in either ear on any one test: CS, monosyllables in noise, gap detection and MLD)	(mean 8 yrs and 3 mos); 5 girls. On the treatment waiting list. Difficulty with SIN task, but N results for competing tasks.	1) SIN: Treatment gr showed improvement in SIN scores post-intervention, but not CG. Improvements were significant for the RE for the noise + dichotic gr and for the LE in the noise gr.<br>2) CS: For the noise + dichotic gr, significant improvement was noted in the LE for short CS and for both ears for the long CS. Some improvement noted for the LE for long CS for the noise gr.<br>No change noted for the CG for both measures.	

REVIEW ARTICLES					
**Authors/7r/ country**	**Aim of review**	**Study design**	**Inclusion/exclusion criteria for the articles**	**Key findings**	**Efficacy**
Reynolds et al., 2016/ USA [43]	Academic outcomes of personal FM system (binaural fitting) use at school.	Systematic review	In all studies:<br>- Personal FM devices, fitted binaurally used at school.<br>Studies inclusion criteria:<br>- Published from January 2003 to March 2014, written in English;<br>- **Population**: Children and adolescent aged 5 to 21 yrs old, with APD or suspected APD, absence of HL. Presence of ASD, ADHD and learning disability was included;<br>- **Treatment**: Use of FM system for at least 4 wks;<br>- **Must assess academic outcomes**: improved sound discrimination in class, reading scores, academic performance, phonological awareness, speech perception, attention.<br>- Description of assessment to measure outcomes.<br>- Statistics to measure change in performance.<br>- Level of evidence: III or better.<br>Exclusion criteria:<br>- Nonelectronic source of literature.<br>- Children with cochlear implants, HA or other corrective devices.<br>- Infrared sound field systems.<br>- Case-series design, poor-quality cohort, case-control design, expert opinion.	7 articles were included (one RCT and 6 quasi-experimental).<br>Some studies reported regular monitoring.<br>Population: 211 participants in all included studies. 118 had APD primarily, 38 had dyslexia, 17 had ASD, 10 had Friedreich's ataxia and 4 had ADHD. Age range: 7 to 15 yrs old.<br>Outcomes:<br>5 studies demonstrated statistically significant improvement in speech perception and recognition with FM use. Improvement noted in 3 studies in listening and attention abilities. Academic performance showed improvement in 5 studies in reading, sentence recall, working memory, best learning and retention of information. Parents and teacher noted changes in academic performance after FM use.<br>The only two studies that met the current scoping review's inclusion criteria were the studies from Umat et al. (2011) and from Sharma et al. (2012) which are already discussed.	Moderate evidence found to suggest the use of a personal FM system for children with AP difficulties. It may improve academic outcomes, especially related speech perception and recognition in the classroom and classroom listening behaviours.<br>Effect size, N/A.
Wilson et al., 2013/ Australia	Impact of AT or language	Systematic review without	Studies inclusion criteria:<br>- Auditory training study (auditory and spoken language intervention studies were included in this review).	7 studies were included. 3 studies were published after 2006, but only 1 was included in the current scoping review. Age range of the participants was 6 to 16 yrs old.	P300 may be sensitive to change following

[44]	training on AEP.	meta-analysis	- Participants may or may not have a diagnosis of APD consistent with ASHA (2005) and AAA (2010), but AP skills must have been assessed before training.<br>- Examine whether trainees' symptoms significantly improve after training.<br>- May include a CG (tested on the same pre-training and post-training measures).<br>- Statistical methods to compare pre-post treatment measures within and/or between the treatment and CG.<br>- No limit placed on the presence or absence of comorbid disorders such as specific language impairment, specific reading disorder, ASD, or ADHD.<br>- Treatment outcomes were measured electrophysiologically using any auditory evoked potential/s such as those listed by ASHA.	P300 (Alsonso and Schochat, 2009)<br>Latency of P300 evoked by tone-bursts may be sensitive to behavioural changes in AP following AT.<br>Improvement noted in SSI-ICM, SIN, SSW and NVDA scores post-intervention.	AT in children with APD.<br>Effect size, N/A.

AAA = American Academy of Audiology; ABR = auditory brainstem response; ACPT = Auditory Continuous Performance Test; ADHD = Attention Deficit/Hyperactivity Disorder; AEP = auditory evoked potential; AFG = auditory figure ground; AFT = Auditory Fusion Test; ALLR = auditory long latency response; AN = abnormal; AP = auditory processing; APD = auditory processing disorder; ART = acoustic reflex threshold; ASD = autism spectrum disorder; ASHA = American Speech-Language-Hearing Association; AT = auditory training; AWMA = Automated Working Memory Assessment; BIC = binaural interaction component; BKB SIN = Bamford-Kowal-Bench Speech-in-Noise Test; BLD = bilateral deficit; CASL = Comprehensive Assessment of Spoken Language; CCC-2 = Children's Communication Checklist-Second Edition; CELF-4 = Clinical Evaluation of Language Fundamentals® - Fourth Edition; CG = control group; CHAPS = Children's Auditory Performance Scale; CMAA = concurrent minimum audible angle; CNS = central nervous system; CRW = compressed and reverberated words; CW or CWT = Competing Words Test; CS or CST = Competing Sentences Test; (D)DDT = (Double) Dichotic Digits Test; DAP = dichotic auditory processing; DAT = dichotic auditory training; DCV = dichotic consonant vowel; DEC = decoding; DIID = differential interaural intensity difference; DITD = dichotic interaural time difference; DLD = dichotic listening deficits; DOT = dichotic offset training; DPOAE = distortion product otoacoustic emissions; DPT = Duration Pattern Test; FAPC = Fisher's Auditory Problem Checklist; FM = frequency modulation; FPT = Frequency Pattern Test; FW(T) = Filtered Words (Test); GDT = Gap Detection Test; GIN = Gaps in Noise Test; gr. or Gr. = group; HA = hearing aid(s); hr = hour; HINT = Hearing in Noise Test; IQ = intelligence quotient; INT = integration; IVA-CPT = Integrated Visual and Auditory Continuous Performance Test; LEB = Logiciel d'écoute dans le bruit; LC = left competitive; LE = left ear; LED = left ear deficit; LIFE = Listening Inventory for Education; LIFE-R = Listening Inventory for Education – Revised; LIFE-UK = Listening Inventory for Education-United Kingdom Edition; LiSN = Listening in Spatialized Noise; LiSN-S = Listening in Spatialized Noise (sentence test); LPFS = low-pass filtered speech; MAPA = Multiple Auditory Processing Assessment; MHAVIE = Mesure des habitudes de vie; MLD = masking level difference; mo/mos = month(s); MLR = middle latency response; mSAAT = Monaural Selective Auditory Attention Test; N = normal; NH = normal hearing; NR = noise reduction; NVDA = dichotic nonverbal directed attention; NVDT = Nonverbal Dichotic Test; OM = otitis media; ORG = organization; PCST = Persian Competing Sentences Test; PCWT = Persian Competing Words Test; PE = pressure equalization; PER = Programa de Escuta no Ruído; P-PST = Persian Phonemic Synthesis Test; PPST or PPT = Pitch Pattern Sequence Test or Pitch Pattern Test; PMR = Progressive Matrices of Raven et al.; PRDDT or RDDT = Persian Randomized Dichotic Digit Test; PTA = puretone average; QUIL = Quick Interactive Language Screener; Quick SIN = Quick Speech-in-Noise Test; RAMST = Revised Auditory Memory and Sequencing Test; RAVLT = Rey Auditory Verbal Learning Test; REA = right ear advantage; RE = right ear; RGDT = Random Gap Detection Test; RNC = right non-competitive; SAB = Scale of Auditory Behaviour; SAT = School Achievement Test; SCAN-C/A = Test for Auditory Processing Disorders in Children-Revised/Adolescents and Adults; SCANA = Test for Auditory Processing Disorder; SCAP = Screening Checklist for Auditory Processing; SD = standard deviation; SIFTER = Screening Instrument For Targeting Educational Risk; SIN = Speech-in-Noise; SNR = signal-to-noise ratio; SPD = spatial processing disorder; SPIN = Speech-in-Noise Test; SPIN-IE = Speech-in-Noise Test (Indian English); SSD = single subject design; SSI-ICM = Synthetic Sentence Identification with Ipsilateral Competitive Message; SSN = speech in spatial noise; SSQ = Speech, Spatial and Qualities of Hearing Scale questionnaire; SSW = Staggered Spondaic Word Test; SRT = speech reception thresholds; STM = spectro-temporal modulation; SW = synthetic words; TAPS-3 = Test of Auditory Processing Skills; TEACh = Test of Everyday Attention for Children; TEAP = Teacher Evaluation of Auditory Performance; TONI = Test of Nonverbal Intelligence; TFM = tolerance-fading Memory; TD = typically developing; TONI-3 or 4 = Test of Nonverbal Intelligence Third or Fourth Edition; TOVA = Test of Variables of Attention—Auditory; UK = United Kingdom; USA = United States of America; WARP = Wheldall Assessment of Reading Passages; WISC = Wechsler Intelligence Scale for Children; WIN = Word in Noise Test; WM = working memory; WNL = within normal limits; WNV = Wechsler Nonverbal; WRS = word recognition score; WSS = word sounded similar; yr/yrs = year(s).

References

[1] S. Ahmed, S. Tawfik, M. Bakr, E. Abdelhaleem, and E. Mohamed, “Remediation of central auditory processing disorders in children with learning disability: a comparative study,” *J Curr Med Res Pract*, vol. 1, no. 3, p. 86, 2016, doi: 10.4103/2357-0121.199359.

[2] S. M. Oraky, S. Tawfik, M. Salama, and E. S. Mohamed, “Comparing outcome of formal and informal remediation programs in children with central auditory processing disorder,” *Egyptian Journal of Otolaryngology*, vol. 33, no. 2, pp. 502–507, 2017, doi: 10.4103/ejo.ejo_3_17.

[3] C. Donadon, M. D. Sanfins, L. R. Borges, and M. F. Colella-Santos, “Auditory training: Effects on auditory abilities in children with history of otitis media,” *International Journal of Pediatric Otorhinolaryngology*, vol. 118, pp. 177–180, 2019, doi: 10.1016/j.ijporl.2019.01.002.

[4] R. Filippini, D. M. Befi-Lopes, and E. Schochat, “Efficacy of Auditory Training Using the Auditory Brainstem Response to Complex Sounds: Auditory Processing Disorder and Specific Language Impairment,” *Folia Phoniatrica Et Logopaedica*, vol. 64, no. 5, pp. 217–226, 2012, doi: 10.1159/000342139.

[5] E. Schochat, F. E. Musiek, R. Alonso, and J. Ogata, “Effect of auditory training on the middle latency response in children with (central) auditory processing disorder.,” *Brazilian journal of medical and biological research = Revista brasileira de pesquisas medicas e biologicas*, vol. 43, no. 8, pp. 777–85, 2010.

[6] Â. De Melo, C. L. Mezzomo, M. V. Garcia, and E. P. V. Biaggio, “Computerized auditory training in students: Electrophysiological and subjective analysis of therapeutic effectiveness,” *International Archives of Otorhinolaryngology*, vol. 22, no. 1, pp. 23–32, 2018, doi: 10.1055/s-0037-1600121.

[7] M. D. Barker and T. J. Bellis, “Effectiveness of a Novel Computer/Tablet-Based Auditory Training Program in Improving Dichotic Listening Skills in Children,” *J Speech Pathol Ther*, vol. 03, no. 01, 2018, doi: 10.4172/2472-5005.1000129.

[8] M. Delphi and F. Zamiri Abdollahi, “Dichotic training in children with auditory processing disorder,” *Int J Pediatr Otorhinolaryngol*, vol. 110, pp. 114–117, Jul. 2018, doi: 10.1016/j.ijporl.2018.05.014.

[9] M. E. Mahdavi, M. Rezaeian, H. Zarrinkoob, M. Rezaeian, and A. Akbarzadeh, “Effect of a Dichotic Interaural Time Difference Program on Dichotic Listening Deficit of Children with Learning Difficulty,” *J Am Acad Audiol*, vol. 32, no. 05, pp. 295–302, May 2021, doi: 10.1055/s-0041-1728753.

[10] Moncrieff DW and Wertz D, “Auditory rehabilitation for interaural asymmetry: preliminary evidence of improved dichotic listening performance following intensive training.,” *International Journal of Audiology*, vol. 47, no. 2, pp. 84–97, février 2008, doi: 10.1080/14992020701770835.

[11] A. Nazeri, H. Bagheri, A. A. Baghban, and E. Negin, “Comparison of Persian staggered spondaic word test’s scores before and after rehabilitation in children with amblyaudia.,” *Auditory & Vestibular Research (2423-480X)*, vol. 29, no. 3, pp. 165–171, juillet 2020.

[12] S. S. Shoemaker, “The impact of Dichotic Auditory Training in children.,” *Dissertation Abstracts International: Section B: The Sciences and Engineering*, vol. 71, no. 6-B, p. 3567, 2010.

[13] K. E. Stephenson, “Effects of dichotic auditory training on children with central auditory processing disorder.,” *Dissertation Abstracts International: Section B: The Sciences and Engineering*, vol. 69, no. 3-B, p. 1542, 2008.

[14] Y. Lotfi, A. Moosavi, F. Z. Abdollahi, E. Bakhshi, and H. Sadjedi, “Effects of an Auditory Lateralization Training in Children Suspected to Central Auditory Processing Disorder,” *Journal of Audiology and Otology*, vol. 20, no. 2, pp. 102–108, Sep. 2016, doi: 10.7874/jao.2016.20.2.102.

[15] Y. Lotfi, A. Moosavi, F. Zamiri Abdollahi, and E. Bakhshi, “Auditory Lateralization Training Effects on Binaural Interaction Component of Middle Latency Response in Children Suspected to Central Auditory Processing Disorder,” *Indian Journal of Otolaryngology and Head and Neck Surgery*, vol. 71, no. 1, pp. 104–108, 2019, doi: 10.1007/s12070-018-1263-1.

[16] A. Koravand, E. Parkes, F. Duquette-Laplante, C. Bursch, and S. Tomaszewski, “The effects of singing lessons on speech evoked brainstem responses in children with central auditory processing disorders,” *Canadian Acoustics - Acoustique Canadienne*, vol. 47, no. 2, pp. 31–40, 2019.

[17] D. Tomlin and A. Vandali, “Efficacy of a deficit specific auditory training program for remediation of temporal patterning deficits.,” *International Journal of Audiology*, vol. 58, no. 7, pp. 393–400, juillet 2019, doi: 10.1080/14992027.2019.1585586.

[18] E. Negin, G. Mohammadkhani, S. Jalaie, and F. Jarollahi, “Efficacy of phonemic training program in rehabilitation of Persian-speaking children with auditory processing disorder: a single subject study,” *AVR*, pp. 116–125, Sep. 2018, doi: 10.18502/avr.v27i3.52.

[19] S. Cameron and H. Dillon, “Development and Evaluation of the LiSN & Learn Auditory Training Software for Deficit-Specific Remediation of Binaural Processing Deficits in Children: Preliminary Findings,” *Journal of the American Academy of Audiology*, vol. 22, no. 10, pp. 678–696, Dec. 2011, doi: 10.3766/jaaa.22.10.6.

[20] S. Cameron, H. Glyde, and H. Dillon, “Efficacy of the LiSN & Learn auditory training software: randomized blinded controlled study,” *Audiology Research*, vol. 2, no. 1, pp. 86–93, 2012, doi: 10.4081/audiores.2012.e15.

[21] K. Graydon, B. Van Dun, D. Tomlin, R. Dowell, and G. Rance, “Remediation of spatial processing disorder (SPD).,” *International Journal of Audiology*, vol. 57, no. 5, pp. 376–384, mai 2018, doi: 10.1080/14992027.2018.1431403.

[22] P. D. Brasil and E. Schochat, “Efficacy of auditory training using the Programa de Escuta no Ruido (PER) software in students with auditory processing disorders and poor school performance.,” *CoDAS*, vol. 30, no. 5, p. e20170227, 2018, doi: 10.1590/2317-1782/20182017227.

[23] B. Jutras, M. Owliaey, M. Gagnon, and C. Phoenix, “Impact de l’entraînement auditif sur les habiletés d’écoute dans le bruit des enfants ayant un trouble de traitement auditif : résultats d’une étude pilote.,” *Canadian Journal of Speech-Language Pathology & Audiology*, vol. 39, no. 4, pp. 346–361, 2015.

[24] B. Jutras, L. Lafontaine, M.-P. East, and M. Noël, “Listening in noise training in children with auditory processing disorder: exploring group and individual data,” *Disability and Rehabilitation*, vol. 41, no. 24, pp. 2918–2926, Nov. 2019, doi: 10.1080/09638288.2018.1482377.

[25] H. Y. J. Loo, "Management of children with auditory processing disorder," University of London, University College London (United Kingdom), Ann Arbor, 2012. [Online]. Available: https://login.proxy.bib.uottawa.ca/login?url=https://www.proquest.com/dissertations-theses/management-children-with-auditory-processing/docview/1512406809/se-2?accountid=14701

[26] J. H. Y. Loo, S. Rosen, and D.-E. Bamiou, "Auditory Training Effects on the Listening Skills of Children With Auditory Processing Disorder.," *Ear and hearing*, vol. 37, no. 1, pp. 38–47, 2016, doi: 10.1097/AUD.0000000000000225.

[27] M. R. Hassaan and O. A. Ibraheem, "Auditory training program for Arabic-speaking children with auditory figure-ground deficits," *International Journal of Pediatric Otorhinolaryngology*, vol. 83, no. 4, pp. 160–167, Apr. 2016, doi: 10.1016/j.ijporl.2016.02.003.

[28] P. Jalilzadeh Afshari *et al.*, "Auditory Spectro-Temporal Modulation Training in Children With Auditory Processing Disorder," *IRJ*, vol. 20, no. 2, pp. 185–198, Jun. 2022, doi: 10.32598/irj.20.2.1392.2.

[29] P. Kumar, N. K. Singh, and R. O. Hussain, "Effect of speech in noise training in the auditory and cognitive skills in children with auditory processing disorders.," *International journal of pediatric otorhinolaryngology*, vol. 146, no. gs2, 8003603, p. 110735, 2021, doi: 10.1016/j.ijporl.2021.110735.

[30] P. Kumar, N. K. Singh, and R. O. Hussain, "Efficacy of Computer-Based Noise Desensitization Training in Children With Speech-in-Noise Deficits.," *American journal of audiology*, no. dgg, 9114917, pp. 1–16, 2021, doi: 10.1044/2021_AJA-20-00153.

[31] A. R. Maggu and A. Yathiraj, "Effect of Noise Desensitization Training on Children with Poor Speech-In-Noise Scores.," *Canadian Journal of Speech-Language Pathology & Audiology*, vol. 35, no. 1, pp. 56–63, 2011.

[32] A. Maggu and A. Yathiraj, "Effect of temporal pattern training on specific central auditory processes," *Dissertation AIISH, Mysore*, vol. 9, p. 11, 2011 2010.

[33] G. Stavrinos, "Effects of Remote Microphone Hearing Aids (RMHAs) on listening-in-noise, attention and memory in school-aged children with A," University of London, University College London (United Kingdom), Ann Arbor, 2019. [Online]. Available: https://login.proxy.bib.uottawa.ca/login?url=https://www.proquest.com/dissertations-theses/effects-remote-microphone-hearing-aids-rmhas-on/docview/2307392189/se-2?accountid=14701

[34] G. Stavrinos, V. V. Iliadou, M. Pavlou, and D.-E. Bamiou, "Remote Microphone Hearing Aid Use Improves Classroom Listening, Without Adverse Effects on Spatial Listening and Attention Skills, in Children With Auditory Processing Disorder: A Randomised Controlled Trial.," *Frontiers in neuroscience*, vol. 14, no. 101478481, p. 904, 2020, doi: 10.3389/fnins.2020.00904.

[35] C. Umat, S. Z. Mukari, N. F. Ezan, and N. C. Din, "Changes in auditory memory performance following the use of frequency-modulated system in children with suspected auditory processing disorders," *Saudi Medical Journal*, vol. 32, no. 8, pp. 818–824, Aug. 2011.

[36] J. L. Smart, S. C. Purdy, and A. S. Kelly, "Impact of Personal Frequency Modulation Systems on Behavioral and Cortical Auditory Evoked Potential Measures of Auditory Processing and Classroom Listening in School-Aged Children with Auditory Processing Disorder.," *Journal of the American Academy of Audiology*, vol. 29, no. 7, pp. 568–586, Aug. 2018, doi: 10.3766/jaaa.16074.

[37] F. Kuk, A. Jackson, D. Keenan, and C. Lau, "Personal Amplification for School-Age Children with Auditory Processing Disorders," *J Am Acad Audiol*, vol. 19, no. 06, pp. 465–480, Jun. 2008, doi: 10.3766/jaaa.19.6.3.

[38] A. Moossavi, S. Mehrkian, Y. Lotfi, S. Faghihzadeh, and H. Sadjedi, "The effect of working memory training on auditory stream segregation in auditory processing disorders children," *Iranian Rehabilitation Journal*, vol. 13, no. 1, pp. 22–27, 2015.

[39] Bellis TJ and Anzalone AM, "Intervention approaches for individuals with (central) auditory processing disorder.," *Contemporary Issues in Communication Science & Disorders*, vol. 35, pp. 143–153, 2008, doi: 10.1044/cicsd_35_f_143.

[40] M. Sharma, S. C. Purdy, and A. S. Kelly, "A randomized control trial of interventions in school-aged children with auditory processing disorders.," *International Journal of Audiology*, vol. 51, no. 7, pp. 506–518, juillet 2012, doi: 10.3109/14992027.2012.670272.

[41] M. Sharma, S. Purdy, and A. Kelly, "The Contribution of Speech-Evoked Cortical Auditory Evoked Potentials to the Diagnosis and Measurement of Intervention Outcomes in Children with Auditory Processing Disorder," *Semin Hear*, vol. 35, no. 01, pp. 051–064, Jan. 2014, doi: 10.1055/s-0033-1363524.

[42] H. Putter-Katz, L. Adi-Bensaid, I. Feldman, and M. Hildesheimer, "Effects of speech in noise and dichotic listening intervention programs on central auditory processing disorders.," *Journal of basic and clinical physiology and pharmacology*, vol. 19, no. 3–4, pp. 301–16, 2008.

[43] S. Reynolds, H. M. Kuhaneck, and B. Pfeiffer, "Systematic Review of the Effectiveness of Frequency Modulation Devices in Improving Academic Outcomes in Children With Auditory Processing Difficulties.," *American Journal of Occupational Therapy*, vol. 70, no. 1, pp. 1–12, Feb. 2016, doi: 10.5014/ajot.2016.016832.

[44] W. J. Wilson, W. Arnott, and C. Henning, "A systematic review of electrophysiological outcomes following auditory training in school-age children with auditory processing deficits.," *International Journal of Audiology*, vol. 52, no. 11, pp. 721–730, Nov. 2013, doi: 10.3109/14992027.2013.809484.
